# TRAF2 Controls Death Receptor-Induced Caspase-8 Processing and Facilitates Proinflammatory Signaling

**DOI:** 10.3389/fimmu.2019.02024

**Published:** 2019-08-29

**Authors:** Jennifer Kreckel, Mohammed A. Anany, Daniela Siegmund, Harald Wajant

**Affiliations:** ^1^Division of Molecular Internal Medicine, Department of Internal Medicine II, University Hospital Würzburg, Würzburg, Germany; ^2^Division of Genetic Engineering and Biotechnology, Department of Microbial Biotechnology, National Research Centre, Giza, Egypt

**Keywords:** caspase-8, death receptors, CD95, TNFR1, TRAF1, TRAF2, TRAILR1, TRAILR2

## Abstract

Tumor necrosis factor (TNF) receptor associated factor-2 (TRAF2) knockout (KO) cells were generated to investigate the role of TRAF2 in signaling by TNFR1 and the CD95-type death receptors (DRs) TRAILR1/2 and CD95. To prevent negative selection effects arising from the increased cell death sensitivity of TRAF2-deficient cells, cell lines were used for the generation of the TRAF2 KO variants that were protected from DR-induced apoptosis downstream of caspase-8 activation. As already described in the literature, TRAF2 KO cells displayed enhanced constitutive alternative NFκB signaling and reduced TNFR1-induced activation of the classical NFκB pathway. There was furthermore a significant but only partial reduction in CD95-type DR-induced upregulation of the proinflammatory NFκB-regulated cytokine interleukin-8 (IL8), which could be reversed by reexpression of TRAF2. In contrast, expression of the TRAF2-related TRAF1 protein failed to functionally restore TRAF2 deficiency. TRAF2 deficiency resulted furthermore in enhanced procaspase-8 processing by DRs, but this surprisingly came along with a reduction in net caspase-8 activity. In sum, our data argue for (i) a non-obligate promoting function of TRAF2 in proinflammatory DR signaling and (ii) a yet unrecognized stabilizing effect of TRAF2 on caspase-8 activity.

## Introduction

The death domain (DD)-containing receptors (DRs) of the tumor necrosis factor (TNF) receptor superfamily (TNFRSF) can be subdivided into three groups according to how DD-containing signaling proteins and caspase-8 are used to induce apoptosis (1). The receptors of the CD95-type DR group (CD95) (Apo1, Fas), TNF-related apoptosis inducing ligand (TRAIL) receptor-1 (TRAILR1, DR4), and TRAILR2 (DR5) recruit procaspase-8 directly in the receptor signaling complex by means of the DD-containing adapter protein Fas-associated death domain (FADD). In contrast, the receptors of the TNFR1-type DR group (TNFR1, DR3) activate the FADD–caspase-8 dyad indirectly in cytosolic protein complexes. The receptors of the third group of DRs (p75NGFR, EDAR, DR6) induce cell death under poorly defined circumstances by heterogeneous mechanisms without obvious involvement of FADD and caspase-8. The CD95- and TNFR1-type DRs and DR6 also have the ability to trigger necroptotic cell death *via* the receptor-interacting protein serine/threonine kinase-1 (RIPK1)–RIPK3 mixed lineage kinase domain-like (MLKL) pathway (2, 3). There are again clearly distinguishable differences in the molecular mechanisms involved. While FADD is essentially required for CD95-type DR-induced necroptosis, TNFR1-induced necroptosis is even enhanced in the absence of FADD (4, 5). The importance of FADD for DR6-induced necroptosis has not been investigated, yet.

DRs of the CD95 and TNFR1 type are not only able to induce cell death but can also trigger proinflammatory signaling pathways leading to the activation of transcriptions factors of the nuclear factor of kappaB (NFκB) family and mitogen-activated protein (MAP) kinases (MAPKs) (1). Again, FADD and caspase-8 are of differential relevance. CD95-type DRs require FADD and caspase-8 for proinflammatory signaling while both molecules are dispensable for proinflammatory signaling by TNFR1-type DRs (1). RIPK1 and the DD-containing adapter protein TNF receptor associated death domain protein (TRADD), however, are crucially involved in TNFR1- and CD95-type DR-induced NFκB signaling. The redundant role of TRADD and RIPK1 in DR-induced classical NFκB signaling points to a crucial role of the adapter protein TRAF2 (6–9). TRAF2 not only directly binds to short peptide motifs within the cytoplasmic tail of receptors of the TRAF-interacting subgroup of the TNFRSF but also interacts with TRADD and RIPK1 (10). In the context of signal transduction of TRAF-interacting receptors, TRAF2 and the TRAF2-interacting E3 ligases cellular inhibitor of apoptosis-1 (cIAP1) and cIAP2 have been strongly implicated in activation of the classical NFκB pathway and the JNK MAPK pathway (10, 11). There is furthermore manifold evidence that TRAF2 in conjunction with the cIAPs promotes proinflammatory signaling and limits apoptotic as well as necroptotic activity of TNFR1-type DRs. TRAF2 has also an anti-necroptotic function in CD95-type DR-induced necroptosis (12, 13). The role of TRAF2 in proinflammatory signaling by CD95-type DRs, however, has been poorly addressed so far. In murine embryonal fibroblasts, TRAF2 was found to be required for JNK signaling but was dispensable for NFκB activation (14–17). In murine A20 cells, however, TRAF2 deficiency resulted in inhibition of TRAIL-induced rapid NFκB signaling, while it was dispensable at a later stage. Noteworthy, the TRAF2-independent late mode of NFκB activation described by Zhang et al. required caspase activation and MEKK1 (18). It has been shown that MEKK1 is cleaved during apoptosis by caspases with the release of a signaling stimulating kinase active fragment (19, 20). Thus, the late mode of NFκB activation presumably represents an apoptosis-associated event without major relevance for CD95-type proinflammatory DR signaling in resistant cells.

Here, we knocked out TRAF2 in HCT116-PI3Kmut and HT1080-Bcl2-TNFR2 cells, which are resistant against DR-induced apoptosis downstream of procaspase-8 processing due to inhibition of Bax/Bak-mediated mitochondrial amplification of the apoptotic caspase-8 activity (16, 21, 22). TRAF2-deficient cells remained DR resistant but displayed reduced proinflammatory DR signaling and an unexpected stabilizing effect of TRAF2 on mature caspase-8.

## Results

### TRAIL-Induced Activation of the Classical NFκB Pathway in HCT116 Cells Does Not Require Caspase-8 Activity

**Figure 1:**
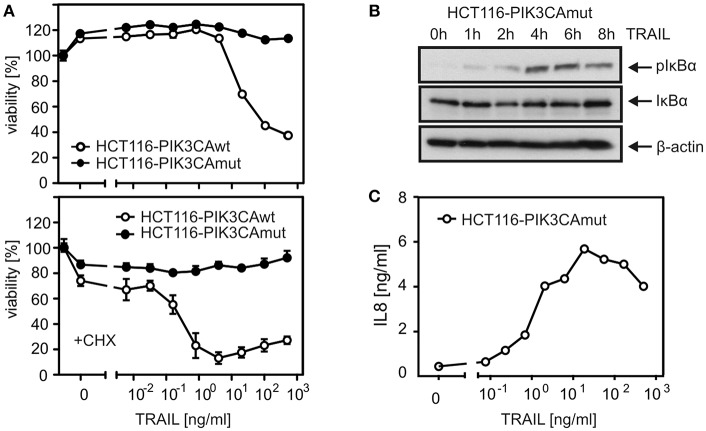
HCT116-PI3Kmut cells are largely protected from DR-induced apoptosis. **(A)** HCT116 cells carrying a single PI3K mut or wt allele were challenged overnight with the indicated concentrations of TRAIL in the absence or presence of 2.5 μg/ml CHX and analyzed for cellular viability. **(B)** Western blot evaluation of phosphorylation and degradation of IκBα in HCT116-PI3Kmut cells stimulated with 100 ng/ml TRAIL. **(C)** Upregulation of IL8 production in HCT116-PI3Kmut cells stimulated overnight with the indicated concentrations of TRAIL.

**Figure 2:**
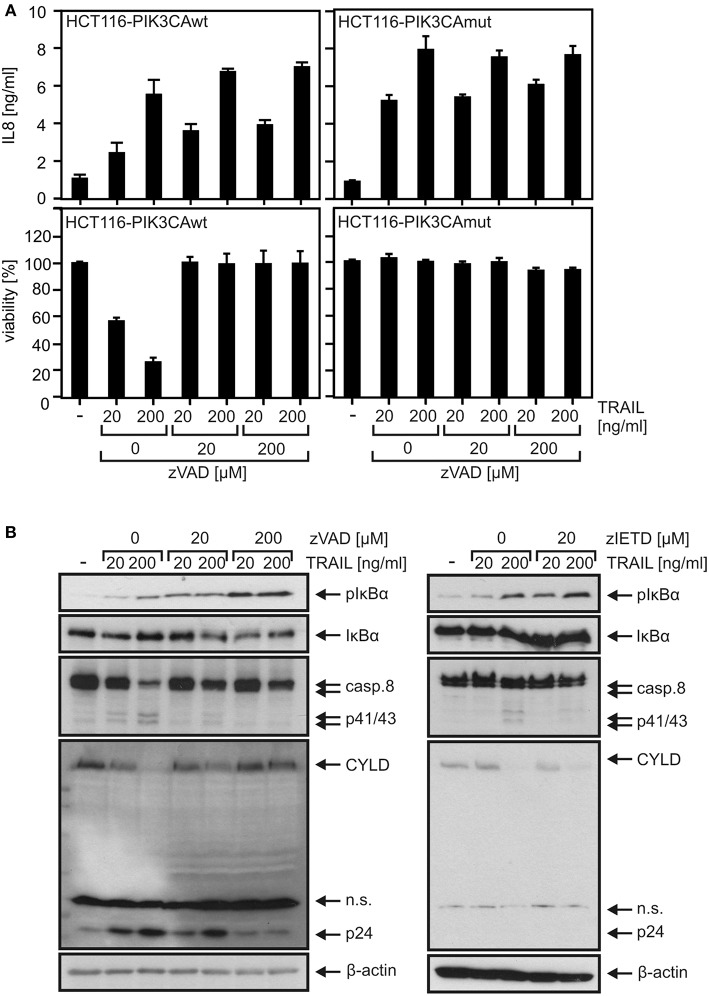
Proinflammatory TRAIL signaling does not require caspase activity in HCT116 cells. **(A)** Apoptosis-sensitive HCT116-PI3Kwt cells (left panel) and apoptosis-resistant HCT116-PI3Kmut cells (right panel) were challenged as indicated with TRAIL and zVAD. The next day, IL8 production and cellular viability were determined. **(B)** HCT116-PI3Kmut cells were stimulated with TRAIL in the presence of zVAD or zIETD for 6 h and were analyzed by Western blotting.

To investigate the relevance of TRAF2 in proinflammatory DR signaling, we knocked out its expression in HCT116-PI3Kmut cells, which express only a mutated active allele of the *PIK3CA* oncogene causing constitutive degradation of Bax (16, 23). The apoptosis-resistant variant was used for two reasons. First, initially, we failed in apoptosis-competent cell lines to obtain TRAF2 knockout (KO) cells with an otherwise well working CRISPR/Cas9 protocol pointing to negative selection of TRAF2 KO cells. Second, since deficiency of TRAF2 typically sensitizes cells for DR-induced apoptosis and as the latter in turn inhibits proinflammatory signaling, TRAF2-deficient apoptosis-resistant cells promise easier dissection of direct effects of TRAF2 deficiency from indirect effects resulting from enhanced cell death sensitivity. In accordance with our previous data (22), HCT116-PI3Kmut cells were largely resistant against TRAIL-induced cell death, even in the presence of CHX as an apoptosis sensitizer, while an HCT116 variant harboring a *PIK3CA* wild-type allele was strongly TRAIL-sensitive (Figure 1A). In accordance with the fact that DR-resistance of HCT116-PI3Kmut cells is due to depletion of Bax, which acts downstream of DR-associated caspase-8 activation, processing of caspase-8 was comparable between HCT116-PI3Kmut and HCT116-PI3Kwt cells (Figure S1A). We furthermore confirmed that TRAIL induces phosphorylation of IκBα and production of the NFκB target interleukin-8 (IL8) in the HCT116-PI3Kmut variant (22) (Figures 1B,C). TRAIL-induced IκBα phosphorylation slowly developed over 1–4 h to reach a plateau lasting several hours (Figure 1B). In view of the controversial reports on the relevance of the enzymatic activity of caspase-8 for CD95-type DR-induced activation of the classical NFκB pathway (1), we analyzed the impact of the pan-caspase inhibitor z-VAD-fmk (ZVAD). While ZVAD completely prevented TRAIL-induced apoptosis in the sensitive HCT116-PI3Kwt variant, it showed no significant effect on TRAIL-induced IκBα phosphorylation in the HCT116-PI3Kmut variant and even enhanced TRAIL-induced IL8 production (Figures 2A,B). In contrast, processing of caspase-8 and processing of the caspase-8 substrate Cyld (24) were fully inhibited by ZVAD (Figure 2B). Similar to ZVAD, the specific caspase-8 inhibitor z-IETD-fmk (zIETD) prevented caspase-8 processing but showed no inhibitory effect on IκBα phosphorylation (Figure 2B). Again, there was also somewhat enhanced TRAIL-induced IL8 production in the HCT116-PI3Kmut cells (Figure S1B). In sum, these data indicate that TRAIL signals classical NFκB activation in HCT116 cells by a caspase-8 activity-independent mode.

### TRAF2 Deficiency Does Not Break Death Receptor Resistance of HCT116-PI3Kmut Cells but Attenuates Proinflammatory Signaling

**Figure 3:**
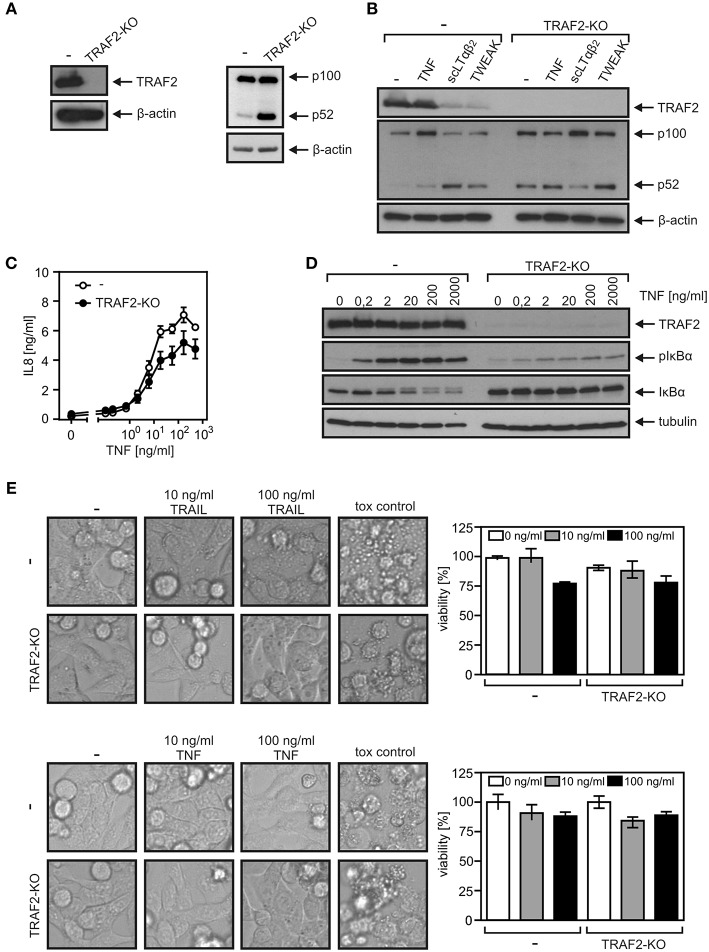
TRAF2-deficient HCT116-PI3Kmut cells remain DR resistant. **(A)** HCT116-PI3Kmut and HCT116-PI3Kmut-TRAF2_KO_ cells were analyzed by Western blotting. **(B)** Cells were stimulated with 200 ng/ml of TNF, LTαβ_2_, or TWEAK or remained untreated. The next day, cells were again analyzed by Western blotting. **(C,D)** Cells were stimulated overnight **(C)** or for 5 min **(D)** with increasing concentrations of TNF. IL8 production **(C)** and phosphorylation and degradation of IκBα **(D)** were analyzed as hallmarks of proinflammatory signaling by ELISA and Western blotting, respectively. **(E)** Cells were challenged overnight with TRAIL or TNF or a cytotoxic cocktail containing 200 ng/ml TNF, 200 ng/ml Fc-CD95L, 200 ng/ml TWEAK, 5 μg/ml CHX, 1 μg/ml Puromycin, and 0.02% sodium azide and were analyzed for viability by microscopic inspection (left panels) or crystal violet staining (right panels).

**Figure 4:**
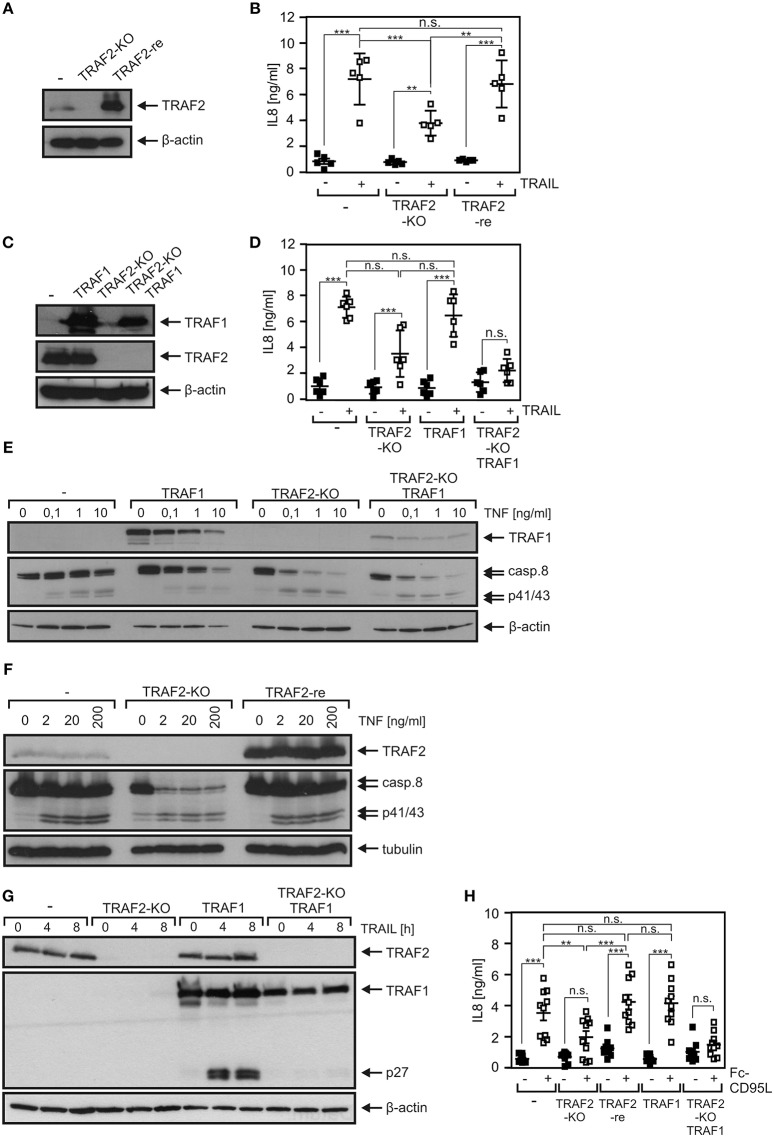
Death receptor-induced IL8 production is attenuated by TRAF2 deficiency and is not restored by TRAF1 expression. **(A)** HCT116-PI3Kmut, HCT116-PI3Kmut-TRAF2_KO_, and HCT116-PI3Kmut-TRAF2_re_ cells were analyzed by Western blotting. **(B)** Cells were stimulated overnight with 200 ng/ml TRAIL and IL8 production was assayed by ELISA. Shown are results from five independent experiments. **(C)** HCT116-PI3Kmut and HCT116-PI3Kmut-TRAF2_KO_ cells along with stable TRAF1 transfectants of these variants were analyzed by Western blotting. **(D)** Cells were analyzed as in “B.” Results are derived from six independent experiments. **(E,F)** Cells were treated with increasing concentrations of TNF for 6 h in the presence of CHX (2.5 μg/ml) and subjected to Western blot analysis. **(G)** CHX-treated cells were stimulated with 200 ng/ml TRAIL for the indicated times and subjected to Western blot evaluation. **(H)** The indicated HCT116-PI3Kmut variants were stimulated overnight with 200 ng/ml Fc-CD95L and IL8 production was again determined by ELISA. Shown are results from 10 independent experiments. ***p < 0.001; **p < 0.01; n.s., not significant.

A TRAF2 KO variant of the HCT116-PI3Kmut cell line was obtained using a CRISPR/Cas9 standard protocol (Figure 3A). In accordance with the crucial role of TRAF2 in the suppression of the alternative NFκB pathway, HCT116-PI3Kmut-TRAF2_KO_ cells showed constitutively enhanced p100 processing, which was not further stimulable by established triggers of the alternative NFκB pathway such as TWEAK and LTαβ_2_ (Figure 3B). Evaluation of TNF-induced phosphorylation and degradation of IκBα and upregulation of IL8 revealed furthermore a non-obligate contribution of TRAF2 to TNF-induced proinflammatory signaling (Figures 3C,D). In particular, despite the broadly documented survival activity of TRAF2 in DR signaling, TRAF2 deficiency had no significant effect on resistance of HCT116-PI3Kmut-TRAF2_KO_ cells against TRAIL- and TNF-induced cell death (Figure 3E). TRAF2 deficiency also resulted in attenuated (~50% reduction) TRAIL-induced production of the NFκB target IL8. Due to the only partial effect of TRAF2 deficiency on DR-induced IL8 production, we confirmed the specificity of the effect by reintroduction of TRAF2. Indeed, HCT116-PI3Kmut-TRAF2_KO_ transfectants stably expressing TRAF2 (HCT116-PI3Kmut-TRAF2_re_) displayed restored IL8 responsiveness (Figures 4A,B). TRAF2 forms heterotrimeric complexes with the closely related TRAF1 protein (25). Moreover, TRAF2–TRAF1 heterotrimers have been found to be superior to TRAF2 homotrimers with respect to binding of cIAP1, which crucially contributes to the activation of the classical NFκB pathway by members of the TNFRSF (25). To evaluate whether TRAF1 enhances TRAIL-induced IL8 production and/or substitutes for TRAF2 in this process, we stably expressed TRAF1 in HCT116-PI3Kmut and HCT116-PI3Kmut-TRAF2_KO_ cells (HCT116-PI3Kmut-TRAF1; HCT116-PI3Kmut-TRAF1-TRAF2_KO_; Figure 4C). Ectopic TRAF1 expression showed no significant effect on TRAIL-induced IL8 production irrespective of the presence of TRAF2 (Figure 4D). Noteworthy, there was enhanced TNF-induced procaspase-8 processing in the absence of TRAF2 (Figures 4E,F), which was fully rescued in the TRAF2-reconstituted cells (Figure 4F). TRAF1 expression levels furthermore dropped down after TNF treatment in the HCT116-PI3Kmut-TRAF1 but not in HCT116-PI3Kmut-TRAF1-TRAF2_KO_ cells (Figure 4E). The latter might reflect the fact that TRAF1 is a substrate of caspase-8 (26, 27). Indeed, using another TRAF1 antibody, we observed significant cleavage of TRAF1 in TRAIL-stimulated HCT116-PI3Kmut-TRAF1 cells (Figure 4G).

**Figure 5:**
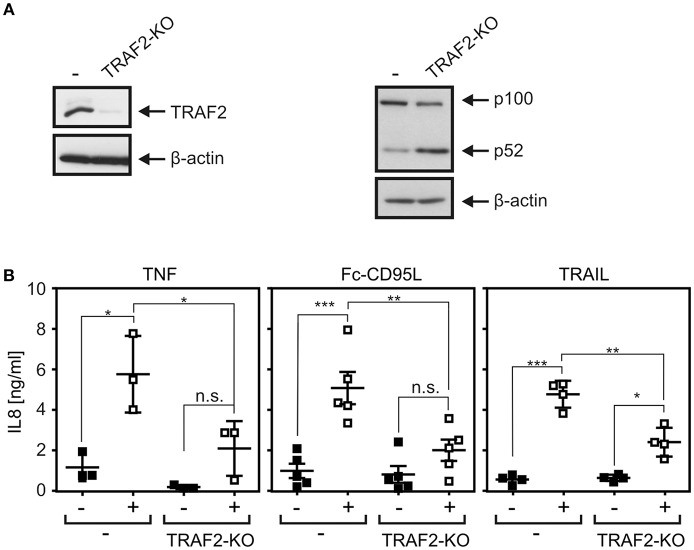
DR-induced IL8 production in TRAF2-deficient HT1080-Bcl2-TNFR2 cells. **(A)** HT1080-Bcl2-TNFR2 and HT1080-Bcl2-TNFR2-TRAF2_KO_ cells were analyzed by Western blotting. **(B)** Cells were stimulated overnight with 200 ng/ml Fc-CD95L, 2 ng/ml TNF, or 200 ng/ml TRAIL and IL8 production was determined by ELISA. Shown are results from three to five independent experiments. ***p < 0.001; **p < 0.01; *p < 0.05; n.s., not significant.

Similar IL8 induction results as observed with TRAIL were furthermore obtained upon stimulation of CD95. Again, IL8 induction was significantly reduced in HCT116-PI3Kmut-TRAF2_KO_ cells and restored to normal levels in the cell variant with reconstituted TRAF2 expression without a significant impact of TRAF1 expression (Figure 4H). To verify the relevance of TRAF2 for CD95-type DR-induced IL8 production in an independent cellular system, we deleted TRAF2 expression in apoptosis-resistant HT1080-Bcl2-TNFR2 cells (Figure 5A). Again, as expected, there was constitutive p100 processing to p52 (Figure 5A). Moreover, there was again significantly reduced IL8 upregulation by the death ligands TNF, Fc-CD95L, and TRAIL (Figure 5B).

### TRAF2 Deficiency Enhanced DR-Induced Caspase-8 Processing

**Figure 6:**
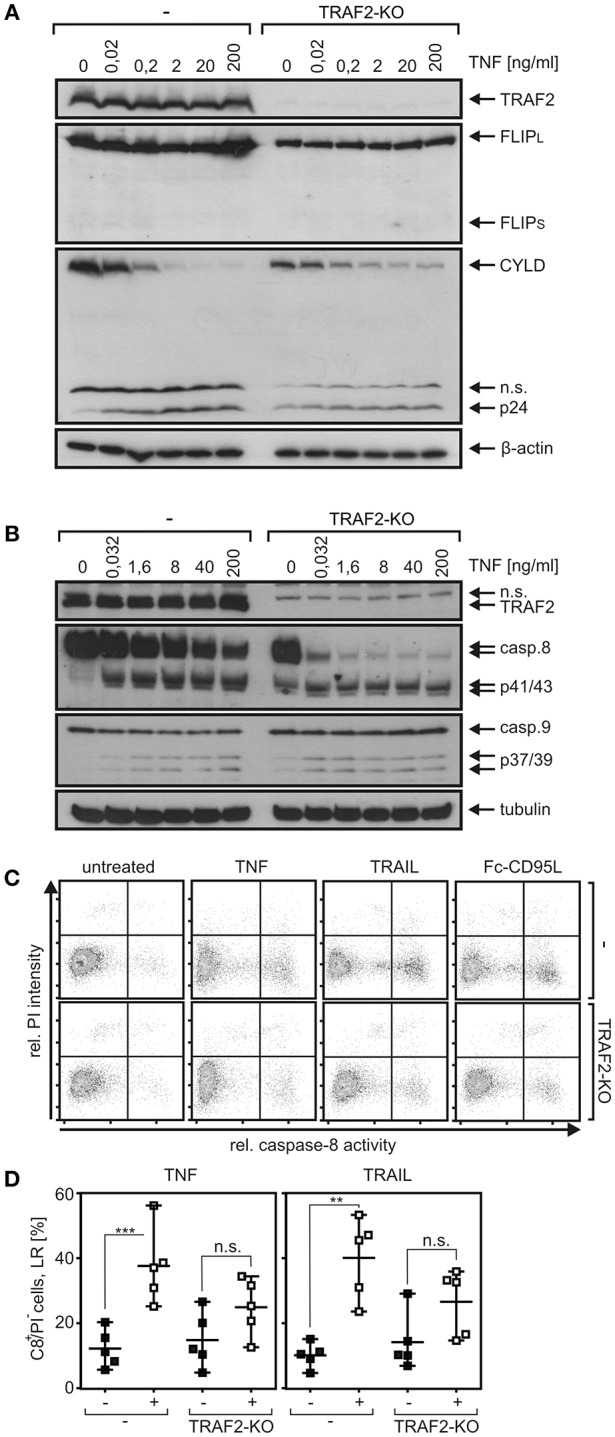
TRAF2 deficiency has no major effect on FLIP expression of HCT116-PI3Kmut cells but enhances TNF-induced procaspase-8 processing. **(A,B)** HCT116-PI3Kmut cells (–) and its TRAF2-deficient variant (TRAF2-KO) were challenged with TNF for 6 h in the presence of 2.5 μg/ml CHX. Total cell lysates were analyzed by Western blotting with respect to the indicated protein species. **(C)** Cells were stimulated with 100 ng/ml TNF, TRAIL, and Fc-CD95L again in the presence of 2.5 μg/ml CHX, and caspase-8 activity was analyzed using the fluorochrome-labeled inhibitor of caspases assay (FLICA) method with FAM-IETD-FMK for caspase-8 detection. **(D)** Independent experiments were performed as described in “**C**” for TNFR1 and the CD95-type DR CD95 for statistical analysis. ***p < 0.001; **p < 0.01; n.s., not significant. Please note **(i)** that cells do not, or only poorly, die [see lack/low processing of effector caspase in **(A)** and PI staining in **(B)**] despite robust caspase-8 activation/processing.

As shown above in Figures 4E,G, TRAIL-induced TRAF1 cleavage was more efficient in the presence than in the absence of TRAF2. This was a bit surprising in view of the frequently reported anti-apoptotic activity of TRAF2 and a previous report describing TRAF2-dependent K48 ubiquitination of active caspase-8 by cullin-3 resulting in proteasomal degradation of active caspase-8 and an attenuated apoptosis response (9). On the other side, it appears possible that in the special case of TRAF1, its interaction with TRAF2 is required to make it available as a substrate for caspase-8. FLIP proteins are presumably the most potent regulators of procaspase-8 processing and interact not only with caspase-8 but also with TRAF2 and TRAF1 (28, 29) Moreover, the long isoform of FLIP (FLIP_L_) can promote the initial processing step of procaspase-8 processing (30, 31), and murine embryonal fibroblasts of TRAF2 KO mice revealed lower FLIP expression due to enhanced FLIP degradation (32). Last but not least, expression of FLIP proteins is controlled, among others, by the classical NFκB pathway (33, 34). In view of the potential complex interplay of FLIP proteins, caspase-8, TRAF2, and NFκB signaling, we evaluated expression of FLIP proteins in HCT116-PI3Kmut and HCT116-PI3Kmut-TRAF2_KO_ cells. While FLIP_L_ was readily detectable by Western blotting, the short isoform FLIP_S_ was not or only poorly expressed (Figure 6A). It turned out that neither TNF (Figure 6A) nor TRAIL (Figure S2A) upregulated FLIP expression in the parental HCT116-PI3Kmut cells and there was also no obvious effect of TRAF2 deficiency on FLIP expression. There was furthermore no major difference in the recruitment of FLIP proteins to the TRAIL death receptors in the HCT116-PI3Kmut-TRAF2_KO_ cells (Figure S2B). This suggests that the enhanced DR-induced processing of procaspase-8 in TRAF2-deficient cells is not due to a change in the expression of FLIP proteins. Moreover, in accordance with earlier reports [e.g., (21, 35, 36)], we found that expression of FLIP_L_, which promotes initial processing of procaspase-8, as well as expression of FLIP_S_, which completely prevents procaspase-8 processing, interfered with TRAIL- and CD95L-induced IL8 production (Figure S2C). Noteworthy, TNF-induced IL8 production remained unaffected by FLIP_L/S_ expression (Figure S2C). Since expression of FLIP proteins only inhibited TRAIL- and CD95L-induced IL8 production and as the two FLIP variants have opposing effects on procaspase-8 processing, it appears unlikely that the general effects of TRAF2 on DR-induced IL8 production and caspase-8 processing are due to deregulated FLIP activity in TRAF2 KO cells.

In the following, we directly analyzed procaspase-8 processing and caspase-8 activity in TRAF2 KO cells. Indeed, as expected for an antiapoptotic function of TRAF2, there was also enhanced procaspase-8 processing in TNF-stimulated HCT116-PI3Kmut-TRAF2_KO_ cells (Figure 6B). Interestingly, however, death-ligand-induced caspase-8 activity, measured using the FLICA method, was reduced in the TRAF2 KO cells (Figures 6C,D). In line with the reduced caspase-8 activity, there was reduced processing of the caspase-8 substrate Cyld in TRAF2-deficient HCT116-PI3Kmut cells (Figure 6A). In accordance with the fact that TRAF2 deficiency has no major effect on the viability of DR-stimulated HCT116-PI3Kmut cells (Figure 2B), there was no increase in propidium iodide staining, which was performed in parallel with the detection of caspase-8 activity (Figure 6C).

### The SMAC Mimetic BV6 Showed No Major Effect on DR-Induced Proinflammatory Signaling but Accelerated Procaspase-8 Processing

**Figure 7:**
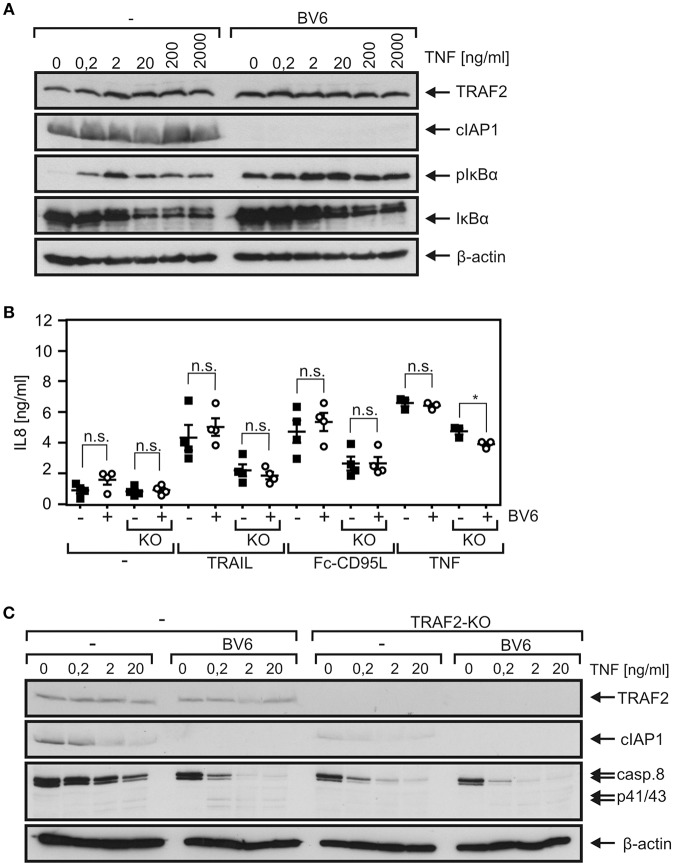
The SMAC mimetic BV6 enhances TNF-induced procaspase-8 processing. **(A)** HCT116-PI3Kmut cells were pretreated or not with 10 μM BV6 for 2 h and were then stimulated with increasing concentrations of TNF for 10 min. Phosphorylation and degradation of IκBα and cIAP1 expression were analyzed by Western blotting. **(B)** HCT116-PI3Kmut (–) and HCT116-PI3Kmut-TRAF2_KO_ (KO) cells were again pretreated with 10 μM for 2 h and then stimulated overnight with 200 ng/ml of TNF, TRAIL, or Fc-CD95L. IL8 production was determined by ELISA. Shown are results of three or four independent experiments. *p < 0.05; n.s., not significant. **(C)** BV6-pretreated (2 h, 10 μM) HCT116-PI3Kmut (–) and HCT116-PI3Kmut-TRAF2_KO_ (TRAF2-KO) cells were stimulated with the indicated concentrations of TNF for 6 h in the presence of 2.5 μg/ml CHX and subjected to Western blot analysis.

TRAF2 fulfills its functions in TNFRSF receptor signaling not least by acting as an adapter to recruit cIAP1 and/or cIAP2. We therefore tested whether treatment of HCT116-PI3Kmut cells with the SMAC mimetic BV6, which triggers proteasomal degradation of cIAP1 and cIAP2 (37), elicits effects similar to TRAF2 deficiency. Expression of cIAP1 was comparable between HCT116-PI3Kmut and HCT116-PI3Kmut-TRAF2_KO_ cells (Figure S3), and cIAP2 was practically not detectable. Treatment with BV6 resulted in a strong reduction of cIAP1 expression in both cell variants. In good accordance with the well-established fact that TRAF2 in concert with cIAP1 and cIAP2 inhibits NIK accumulation and NIK-promoted processing of p100 to p52, BV6 stimulated the latter in HCT116-PI3Kmut but not in HCT116-PI3Kmut-TRAF2_KO_ cells (Figure S3). Instead, there was constitutively enhanced p100 to p52 processing in the HCT116-PI3Kmut-TRAF2_KO_ cells to an extent similar to those induced by BV6 in the parental cell variant. Although there was enhanced steady-state phosphorylation of IκBα phosphorylation in BV6-treated HCT116-PI3Kmut cells, the dose response of TNF-induced IκBα degradation remained largely unaffected (Figure 7A). BV6 treatment showed furthermore no effect on TNF-, TRAIL-, and CD95L-induced IL8 production in HCT116-PI3Kmut cells (Figure 7B). With exception of a minor effect on TNF-induced IL8 production, there was also no effect of BV6 on DR-induced IL8 induction in the HCT116-PI3Kmut-TRAF2_KO_ cells. BV6, however, improved TNF-induced caspase-8 processing in a TRAF2-dependent manner; thus, it enhanced procaspase-8 processing in the HCT116-PI3Kmut cells but not in the TRAF2 KO variant derived thereof (Figure 7C).

## Discussion

Twenty-five years ago, TRAF2 and the TRAF2-interacting E3 ligases cIAP1 and cIAP2 have been identified as components of the TNFR2 signaling complex (38, 39). Afterwards, within a few years, it became evident that TRAF2 (and the cIAPs) recruits directly or indirectly to most, if not all, transmembrane receptors of the TNFRSF. In the context of signaling by receptors of the TNFRSF, TRAF2 has been implicated in the activation of classical NFκB pathways and signaling pathways, resulting in the activation of MAP kinase cascades. Noteworthy, TRAF2, in concert with the cIAPs and TRAF3, triggers the constitutive proteasomal degradation of the MAP3K NIK, the key activator of the alternative NFκB pathway, and thus suppresses the activity of this signaling pathway (10, 11). Recruitment of TRAF2 to receptors of the TNFRSF can deplete the cytosolic pool of TRAF2 molecules by translocation to the plasma membrane and can thus result in activation of the alternative NFκB pathway. Early on, it became evident that TRAF2 also protects cells from TNFR1-induced cell death. Initially, the protective activity of TRAF2 has been attributed to its role in activation of the classical NFκB pathway, but there is growing evidence that it also acts in an NFκB-independent manner by interfering with the cytotoxic activity of caspase-8 and/or RIPK1. Recent studies also point to an anti-necroptotic role of TRAF2 in signaling by DRs of the CD95 type (CD95, TRAILR1, TRAILR2), but the functions of TRAF2 in signal transduction of these death receptors have been poorly addressed so far. We generated TRAF2 KO variants of HCT116-PI3Kmut and HT1080-Bcl2-TNFR2 cells. TRAF2 deficiency showed no major effect on the death receptor resistance of HCT116-PI3Kmut cells. This is in good accordance with the mode of protection in these cells. TRAF2 protects from apoptosis at the level of caspase-8 activation and can thus not bypass the anti-apoptotic effect of the downstream placed Bax deficiency observed in HCT116-PI3Kmut cells. Nevertheless, TRAF2 deficiency and cIAP1 depletion with the SMAC mimetic BV6 come along with enhanced DR-induced processing of procaspase-8 (Figures 6A, 7C). In accordance with the idea that TRAF2 acts in concert with cIAP1 in this scenario, the enhancing effect of BV6 treatment on TNF-induced procaspase-8 processing was abrogated in TRAF2 KO cells (Figure 7C). Surprisingly, enhanced DR-induced procaspase-8 processing in TRAF2 KO cells did not correlate to an increase in net caspase-8 activity. Indeed, there was even a reduction in caspase-8 activity (Figures 6C,D). This might reflect varying specific activities of the different species of enzymatically active caspase-8 (CD95-type DR-bound caspase-8 homodimers, CD95-type DR-bound caspase-8/FLIP_L_ heterodimers, different cytosolic caspase-8 containing complexes etc.) that exist in DR-stimulated cells. This issue must be clarified in future studies. DRs do not trigger necroptosis in HCT116 or HT1080 cells; the anti-necroptotic activity of TRAF2 could therefore not be evaluated in the TRAF2-deficient cells used in this study.

We found furthermore a significant and strong reduction in TNF-induced degradation of IκBα in HCT116-PI3Kmut-TRAF2_KO_ cells (Figure 3D). This correlated with an only slight reduction in TNF-induced upregulation of the NFκB-regulated cytokine IL8 (Figure 3C). The inhibitory effect of TRAF2 deficiency on TNF-induced IL8 production appeared, however, less pronounced at the level of IκBα degradation. This could due to the fact that other TNFR1-induced pathways such as the JNK or p35 pathway contribute to IL8 upregulation, but this appears unlikely as NFκB signaling has practically an obligate function in TNFR1-induced IL8 production. The relatively weak effect of TRAF2 deficiency on TNF-induced IL8 production could also be related to the non-linear and timely complex relationship between IκBα degradation, resynthesis of IκBα, nuclear NFκB activity, and IL8 mRNA synthesis. Thus, the observed reduction in IκBα degradation might be compensated for by diminished and delayed resynthesis of IκBα and a weaker but sustained pathway activity resulting in an only minor inhibitory effect on IL8 production. Indeed, an only partial inhibitory effect of TRAF2 deficiency on TNF-induced NFκB signaling has also been observed in TRAF2-deficient embryonal fibroblasts (6). TRAIL induced IκBα phosphorylation with rather slow kinetics, reaching a plateau after 2–4 h, which lasts for several hours with a hardly detectable effect on net expression levels of IκBα (Figure 1B). Nevertheless, TRAIL-induced IL8 production was in the same range as in the case of TNF (Figures 3C, 4B). TRAIL- and CD95L-induced IL8 production was significantly reduced in TRAF2-deficient cells as well (Figures 4B,D,H, 5B). Although TNF-induced IL8 production appeared a bit less affected by TRAF2 deficiency than TRAIL- and CD95L-induced IL8 production (27 vs. 49% and 45%), this did not result in a significant difference (Figure S4). Noteworthy, although BV6 enhanced TRAIL- and TNF-induced procaspase-8 processing, it had no (TRAIL, CD95L) or only a very minor (TNF in TRAF2 KO cells) effect on IL8 production (Figure 7B). This suggests that reduced DR-induced IL8 production and accelerated caspase-8 processing in TRAF2 KO cells reflect at least two partly different aspects of TRAF2 function in DR signaling. We observed no consistent significant effect of TRAF2 deficiency on net IκBα levels and IκBα phosphorylation in TRAIL- and CD95L-treated cells (data not shown), although there was regularly significantly reduced IL8 production. This could again reflect the overlapping occurrence of degradation and resynthesis of IκBα resulting in a lack of correlation of accumulated active nuclear NFκB transcription factors and net IκBα levels. Slow but long-lasting activation of the classical NFκB pathway by CD95-type DRs has also been observed previously [e.g., (22, 35)] and is in good accordance with the different localization of TRADD, RIPK1, and TRAF2 in TNFR1-type and CD95-type DR signaling. While TRADD, RIPK1, and TRAF2 recruit within seconds to the TNFR1 signaling complex to engage classical NFκB signaling (40), in the context of CD95-type DR signaling, these molecules act in a secondary and delayed formed DR-free cytosolic complex (1). The TRADD–RIPK1 dyad acts upstream of TRAF2 in DR signaling and seems to be obligate for DR-induced IL8 induction (5). TRAF2, however, seems to be dispensable (Figures 4, 5). A possible explanation is that the TRADD–RIPK1 dyad interacts with IKK-stimulating kinases and the IKK complex itself in a TRAF2-independent manner. Indeed, in this respect, there are several possibilities. First, RIPK1 has the ability to directly interact with the NEMO/IKKγ subunit of the IKK complex (41) and also binds the IKK-stimulating kinase MEKK3, which furthermore interacts with TAK1, another IKK-stimulating kinase (42, 43). Second, other members of the TRAF family may substitute for TRAF2 in DR signaling. In fact, a redundant role of TRAF2 and TRAF5 has been reported in murine embryonal fibroblasts derived from TRAF2–TRAF5 double-deficient mice (7). However, we were unable to detect TRAF5 in our cell models and there is no evidence yet in the literature for TRAF2–TRAF5 redundancy in human cells. Since TRAF1 shares many interaction partners with TRAF2, including RIPK1, TRADD, and NEMO/IKKγ (27, 44), and furthermore forms heterotrimeric complexes with TRAF2 (25), we looked for a role of TRAF1 in DR-induced IL8 production. However, while reconstitution of TRAF2 in HCT116-PI3Kmut-TRAF2_KO_ cells restored IL8 induction by death receptors, TRAF1 expression had no effect in these cells (Figure 4). Refined biochemical analysis of DR-induced NFκB-stimulating signaling complexes and generation and evaluation of TRAF2–TRADD and TRAF2–RIPK1 double-deficient cells may help in the future to get a clearer picture on the role of TRAF2 in DR signaling.

## Conclusion

TRAF2 significantly contributes to proinflammatory DR signaling in a manner not replaceable by TRAF1 but has definitely no obligate role in this respect. TRAF2 deficiency enhances TNF-induced procaspase-8 processing but, astonishingly, DR-induced net caspase-8 activity is reduced due to reasons remain to be clarified.

## Materials and Methods

### Reagents and Cell Lines

Human Killer TRAIL (TRAIL) was purchased from Enzo Life Sciences and TNF was a kind gift from Prof. Daniela Männel (University of Regensburg). Fc-CD95L and TWEAK contain an internal Flag tag and were produced by transient transfection of HEK293 cells using PEI (Polysciences Inc., Warrington, USA) as essentially described for other Flag-tagged proteins by Lang et al. (45). Synthesis of BV6 has been described elsewhere (46). Proteins were purified from the supernatant by affinity chromatography on anti-Flag agarose (47). Cycloheximide (CHX), MLN4924, and RPMI1640 were obtained from Sigma-Aldrich (Deisenhofen, Germany), fetal calf serum was from Gibco (Thermo Fisher Scientific, Darmstadt, Germany), z-VAD-fmk was from Bachem (Heidelberg, Germany), and z-IETD-fmk was from BD Biosciences (Heidelberg, Germany). HCT116-*PIK3CA-*wt and HCT116-*PIK3CA-*mut were kindly provided by B. Vogelstein (Johns Hopkins University, Baltimore, USA). HT1080-Bcl2-TNFR2 cells were obtained by retroviral transfection of HT1080-Bcl2(GFP) cells (described in 21) with a mixture of a TNFR2 and a puromycin encoding vector. HCT116-*PIK3CA-*mut cells stably transfected with FLIP_L_ and FLIP_S_ were generated as previously described for HaCaT cells (36).

### Molecular Cloning and Generation of Stable Cell Lines

To generate TRAF2 KO cells, HCT116-PI3Kmut and HT1080-Bcl2-TNFR2 cells were transfected as described below using polyethylenimine (PEI, Polysciences Inc., Warrington, USA) with a mixture of GeneArt CRISPR Nuclease (CD4 Reporter) vector plasmids (ThermoFisher Scientific) encoding two TRAF2-specific guide RNAs and Cas9. The TRAF2 guide sequences (5′ to 3′) used were as follows: CCTGAGCTGCCGGCATTGCGG and GGACCTGGCGATGGCTGACTGG. The guide RNA sequences were chosen from Table 1 of the supplementary website of Mali et al. (48) (http://arep.med.harvard.edu/human_crispr/). Transfected cells were cultivated for 5 days and then seeded at low density to obtain individual clones. The latter was verified for TRAF2 expression by WB to identify clones with successful KO of TRAF2.

The sleeping beauty expression constructs were generated as follows: A synthetic multi-cloning sequence containing sites for *NotI, MluI, NheI, SacII, NruI, SwaI, BglII, XbaI*, and *PacI* flanked by MfeI sites (CAATTGAGCG GCCGCACGCG TGCTAGCCCG CGGGGAGGGT TTGTGGGTGA GTCGCGAGAT TTAAATAGAT CTTCTAGAGT TAATTAACAA TTG) were digested with MfeI and inserted into the plasmid pT2-SVNeo EcoRI site [Addgene, plasmid described in Cui et al. (49)], which is located between two inverted terminal repeats of the sleeping beauty transposon resulting in the vector pT2-SVNeo-MCS. DNA fragments encoding TRAF1 (NCBI Ref. NM AK315476.1) and TRAF2 (NCBI Ref. NM_021138.3) were initially inserted into the vector pTRE-Tight (Clontech, cat. no. 631059) *via* BamHI and NotI (TRAF1) and BamHI and XbaI (TRAF2) to generate pTRE-Tight-TRAF1 and pTRE-Tight-TRAF2. The TRAF1 expression cassette (promoter, coding region, SV40 polyA signal) of pTRE-Tight-TRAF1 was amplified by PCR using primers flanked by SwaI and PacI and inserted into the corresponding sites of pT2-SVNeo-MCS. In addition, we inserted an amplicon containing the tetracycline-controlled transactivator protein tTA-Advanced of the plasmid pTet-Off-Advance (Clontech, cat. no. 630934) *via* the MluI and SwaI sites, resulting in pT2-SVNeo-Tetoff-TRE-TRAF1. The corresponding plasmid pT2-SVNeo-Tetoff-TRE-TRAF2 was obtained by replacing the TRAF1 expression cassette with the corresponding TRAF2 expression cassette of pTRE-Tight-TRAF2 *via* SwaI and PacI. The TRAF1 and TRAF2 encoding pT2-SVNeo plasmids were then used with pCMV-(CAT)T7-SB100 [Addgene, plasmid described in Cui et al. (49)] encoding the sleeping beauty transposon and pT2-SVPuro-CMV-TST206 (kind gift of Thorsten Stühmer, University Hospital Würzburg) encoding a puromycin resistance gene flanked by the sleeping beauty inverted terminal repeats to generate stable TRAF1 and TRAF2 transfectants. For this, cells were transfected chemically using PEI. In brief, 36 μl of a 1 mg/ml solution PEI was mixed with 12 μg of expression vector [60% pT2-SVNeo-Tetoff-TRE-TRAF1 or pT2-SVNeo-Tetoff-TRE-TRAF2, 30% pCMV-(CAT)T7-SB100, pT2-SVPuro-CMV-TST206] and incubated for 15 min at room temperature. The mixture was added to the cells with 15 ml of RPMI1640, and the next day, medium was changed with medium containing 10% FCS and 1 μg/ml puromycin for selection. After 7–10 days, single cells were seeded, expanded, and checked for TRAF1 and TRAF2 expression.

### Viability Assay

Cells (4 × 10^4^ cells/96-well) were challenged with Killer-TRAIL (TRAIL) in triplicate. The next day, cell viability was determined using MTT (3-[4,5-dimethylthiazol-2-yl]-2,5-diphenyl tetrazolium bromide) staining. The optical density (OD) was measured by PHOMO (Anthos), and the values obtained were normalized according to the values of cells receiving no TRAIL (= 100% viability) and cells that have been treated with a cytotoxic mixture containing 200 ng/ml TNF, 200 ng/ml Fc-CD95L, 200 ng/ml TWEAK, 5 μg/ml CHX, 1 μg/ml puromycin, and 0.02% sodium azide (= 0% viability).

### Microscopy

For light microscopy analysis, cells were seeded in 96-well plates and stimulated as described before. Pictures were taken randomly using an Evos X1 microscope.

### ELISA

HCT116-derived cell variants we re seeded with 4 × 10^4^ cells/well, and the HT1080-derived cell variants were seeded with 2 × 10^4^ cells/well. The next day, cell culture medium was replaced to minimize the background caused by constitutive cytokine production before cells were stimulated with the cytokine of interest. After an additional day, cleared supernatants were evaluated for their IL8 content using an IL8 enzyme-linked immunosorbent assay (ELISA) kit (BD Biosciences, Heidelberg, Germany) according to the manufacturer's protocol.

### Western Blot

Cells (2 × 10^6^ per 6-well) were stimulated as indicated in the various figure legends and were scratched on ice. After two washes with ice-cold PBS, total cell lysates were generated by dissolving cells for 5 min at 95°C in Laemmli buffer and sonification (UP100H, Helscher, cycle 1, 100% amplitude, 52 s). Total cell lysates were separated by SDS-PAGE and proteins were transferred to 0.2-μm nitrocellulose blotting membranes (GE Healthcare Life Sciences). Membranes were blocked with 5% non-fat-dried milk in TBS-Tween [0.05% (v/v)], and the proteins of interest were detected with the corresponding primary antibodies (4°C, overnight, TBST) and HRP-linked secondary antibodies (RT, 1–3 h, 2.5% nonfat-dried milk). Antibody complexes were visualized with the ECL solution. Between every step, membranes were washed three times with TBS-Tween [0.05% (v/v)]. The following antibodies were used: anti-caspase-3 (8G10), anti-caspase-9 (#9502), anti-DR4 (D9S1R), anti-DR5 (D4E9), anti-FADD (#2782), anti-phospho-IκBα (Ser32) (14D4), anti-IκBα (L35A5), anti-alpha-Fodrin (#2122), anti-CYLD (D1A10), anti-TRAF1 (45D3), anti-TRAF2 (C192), anti-A20 (D13H3), anti-mouse IgG, HRP-linked antibody (#7076), anti-rat IgG, HRP-linked antibody (#7077) and anti-rabbit IgG, HRP-linked antibody (#7074), which were all from Cell Signaling. Furthermore, anti-PARP (BD Biosciences), HRP-conjugated polyclonal rabbit anti-mouse immunoglobulins (P0260, Dako), anti-caspase-8 (5F7, Enzo), anti-cIAP1 (1E1-1-10, Enzo), anti-tubulin (DM1A, Thermo Fisher Scientific), anti-β-actin (A1978-200 μl, Sigma Aldrich), anti-TRAF1 (H-132; sc-1831; Santa Cruz), anti-FLIP (NF6, AdipoGen), and anti-p52 (05-361, Millipore) have been used.

### Caspase-8 Activity Assay

Cells were cultivated in 12-well plates until they reached a density of approximately 3 × 10^6^ cells per well. Cells were stimulated for 6 h with the indicated reagents and were harvested after a 5-min incubation with trypsin according to the manufacturer's protocol [Application note No. 3021. Rev. 1.3 of NucleoCounter® NC-3000™ (Chemometec)]. Cells were stained using the FAM-FLICA® Caspase-8 Assay Kit (Immunochemistry Technologies; Catalog no. 910). Stained cells were analyzed using NucleoCounter® NC-3000™ software and the values were diagrammed by Graph Pad Prism 5 software.

### Statistical Analysis

Measurements of independent experiments were collected and analyzed using the one-way ANOVA Bonferroni's multiple comparison test function of the GraphPad Prism5 software (GraphPad software, La Jolla, CA, USA).

## Data Availability

The datasets generated for this study are available on request to the corresponding author.

## Author Contributions

JK, MA, and DS performed experiments and prepared figures. JK and HW designed and analyzed the experiments and wrote the manuscript.

### Conflict of Interest Statement

The authors declare that the research was conducted in the absence of any commercial or financial relationships that could be construed as a potential conflict of interest.

## Abbreviations

cIAP1/2: cellular inhibitor of apoptosis-1/2
DR: death receptor
ELISA: enzyme-linked immunosorbent assay
FC: flow cytometry
IL8: interleukin-8
NFκB: nuclear factor kappaB
RIPK1: receptor-interacting serine/threonine kinase 1
TNF: tumor necrosis factor
TNFR1/2: TNF receptor-1/2
TRAF1/2: TNF receptor associated factor-1/2
TRAIL: TNF-related apoptosis inducing ligand
TRAILR1/2: TRAIL receptor-1/2.
