# Supplementary material for: TRAF2 Controls Death Receptor-Induced Caspase-8 Processing and Facilitates Proinflammatory Signaling
Source: Front Immunol. 2019 Aug 29;10:2024. doi: 10.3389/fimmu.2019.02024 (PMC6727177; doi:10.3389/fimmu.2019.02024)
Supplement: Supplementary file 1 [file Data_Sheet_1.pdf]

Supplemental figure 1

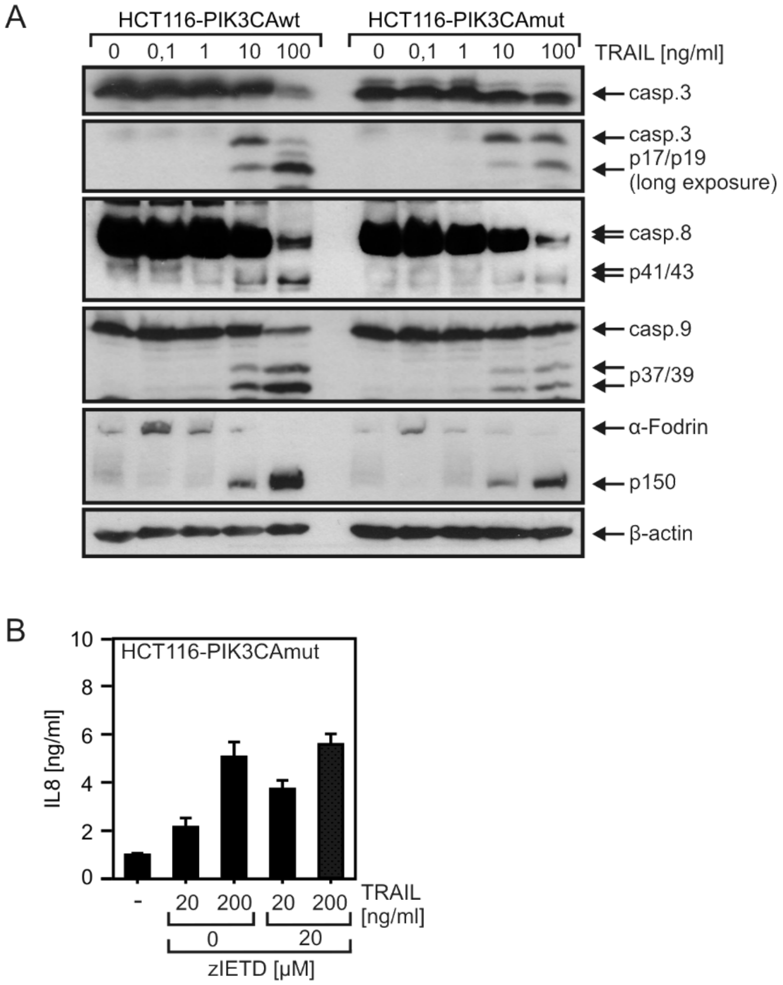

Fig. S1 I (**A**) Cells were stimulated with TRAIL (concentrations as indicated, 18 h) and were analyzed by western blotting for the processing of caspases and caspase substrates. (**B**) HCT116-PI3Kmut cells were stimulated as indicated with TRAIL and zIETD. Next day, IL8 production was determined. Shown is a representative experiment with technical triplicates.

Supplemental figure 2

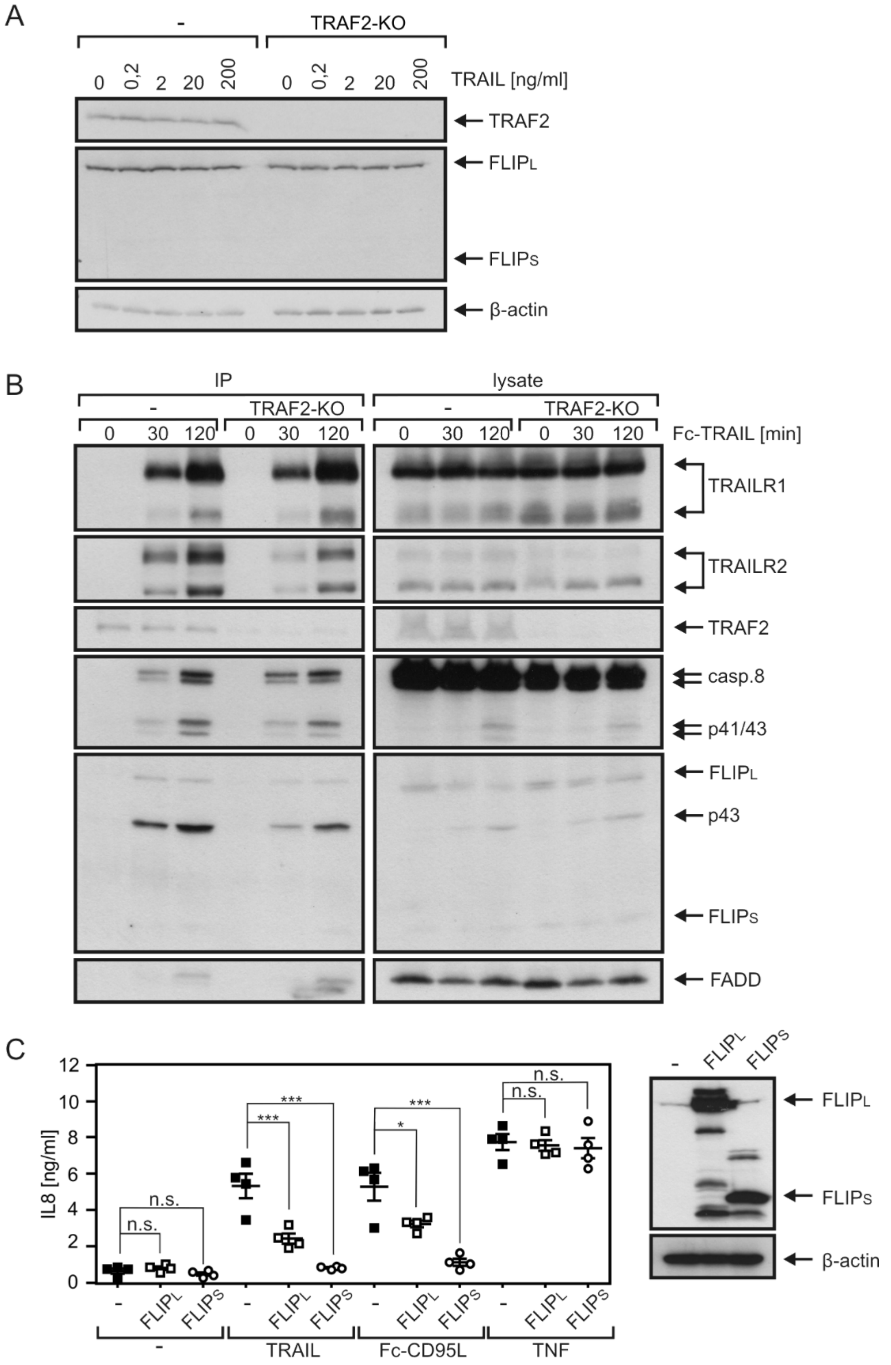

Fig. S2 I (**A**) HCT116-PI3Kmut (-) and HCT116-PI3Kmut-TRAF2$_{KO}$ (TRAF2-KO) cells sensitized with 2.5 µg/ml CHX were stimulated for 6 hours with the indicated concentrations of TRAIL and were then analyzed by western blotting for FLIP expression. (**B**) The TRAIL death receptor signaling complexes were analyzed by co-immunoprecipitation with 1 µg/ml Fc-TRAIL as described elsewhere (Füllsack et al., 2019) and indicated proteins were detected by western blotting. (**C**) HCT116-PI3Kmut cells and transfectants derived thereof stably expressing FLIP$_L$ and FLIP$_S$ were stimulated overnight with 200 ng/ml of TNF, TRAIL or Fc-CD95L. IL8 production was determined by ELISA. Shown are results of 4 independent experiments. "***": $p < 0.001$; "*": $p < 0.05$, "n.s.": not significant.

Füllsack S, Rosenthal A, Wajant H, Siegmund D. Redundant and receptor-specific activities of TRADD, RIPK1 and FADD in death receptor signaling. Cell Death Dis. 2019 Feb 11;10(2):122.

Supplemental figure 3

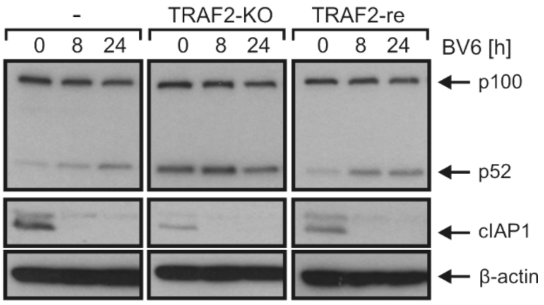

Fig. S3 I HCT116-PI3Kmut (-), HCT116-PI3Kmut-TRAF2$_{KO}$ (TRAF2 KO) and HCT116-PI3Kmut-TRAF2$_{re}$ (TRAF2-re) cells were stimulated for the indicated times with 10 µM BV6 and then subjected to western blotting.

Supplemental figure 4

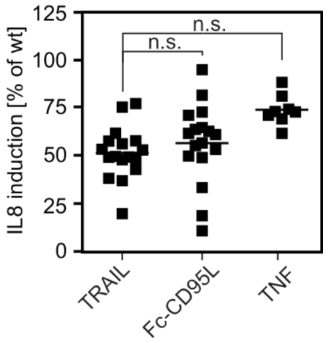

Fig. S4 I We normalized all TRAIL, CD95L- and TNF-induced IL8 values measured in TRAF2 KO cells in the various experiments to the corresponding values in wt cells. This resulted in average of 49 %, 45% and 27% inhibition for TRAIL-, CD95L- and TNF-induced IL8 production. The extent of reduced IL8 production was statistically not different for the three ligand types. “n.s., not significant”.
